# Supplementary material for: NH4+ Toxicity, Which Is Mainly Determined by the High NH4+/K+ Ratio, Is Alleviated by CIPK23 in Arabidopsis
Source: Plants (Basel). 2020 Apr 14;9(4):501. doi: 10.3390/plants9040501 (PMC7238117; doi:10.3390/plants9040501)
Supplement: Supplementary file 1 [file plants-09-00501-s001.zip › Figure S/Table S1.docx]

**Table S1 Plate treatment mediums.**

| Type  Main components | MS | treatment medium ([NH_4_^+^]-[K^+^]) | | |
| --- | --- | --- | --- | --- |
|  |  | 30-0.1 | 10-0.1 | 0-0.1 |
| KNO_3_ (mM) | 19 | -- | -- | -- |
| NH_4_NO_3_ (mM) | 20.6 | 28.75 | 10 | -- |
| NaNO_3_ (mM) | -- | -- | -- | 10 |
| KCl (mM) | -- | 0.1 | 0.1 | 0.1 |
| KH_2_PO_4_ (mM) | 1.25 | -- | -- | -- |
| NaH_2_PO_4_ (mM) | -- | -- | 1.25 | 1.25 |
| NH_4_H_2_PO_4_ (mM) | -- | 1.25 | -- | -- |

Other ions are same with MS, containing 10 g•L^-1^ agar powder and 30 g•L^-1^ sugar. 10 M NaOH was used to adapt pH (5.6-6.0).
