# Supplementary material for: NH4+ Toxicity, Which Is Mainly Determined by the High NH4+/K+ Ratio, Is Alleviated by CIPK23 in Arabidopsis
Source: Plants (Basel). 2020 Apr 14;9(4):501. doi: 10.3390/plants9040501 (PMC7238117; doi:10.3390/plants9040501)
Supplement: Supplementary file 1 [file plants-09-00501-s001.zip › Figure S/Table S2.docx]

**Table S2 Modified 1/5 Hoagland Solution (pH 5.6-6.0).**

| **Name** | **Work concentration (mM)** |
| --- | --- |
| K_2_SO_4_ | Add by design |
| MgSO_4_•7H_2_O | 0.35 |
| NaH_2_PO_4_ | 0.2 |
| H_3_BO_3_ | 12.5×10^-3^ |
| MnSO_4_•H_2_O | 1×10^-3^ |
| CuSO_4_•5H_2_O | 0.5×10^-3^ |
| ZnSO4•7H_2_O | 1×10^-3^ |
| Na_2_MoO_4_•2H_2_O | 0.1×10^-3^ |
| EDTA-Fe | 0.01 |
| Ca(NO_3_)_2_·4H_2_O | 1.4 |
| NH_4_Cl | Add by design |
| CaCl_2_·2H_2_O | 0.15 |

K_2_SO_4_ and NH_4_Cl was added to adjust the level of K^+^ and NH_4_^+^, respectively.
