# Supplementary material for: NH4+ Toxicity, Which Is Mainly Determined by the High NH4+/K+ Ratio, Is Alleviated by CIPK23 in Arabidopsis
Source: Plants (Basel). 2020 Apr 14;9(4):501. doi: 10.3390/plants9040501 (PMC7238117; doi:10.3390/plants9040501)
Supplement: Supplementary file 1 [file plants-09-00501-s001.zip › Figure S/Table S3.docx]

**Table S3 Primers used in this paper.**

| **Primer name** | **Sequence（5’-3’）** | **Gene ID and Annotation** |
| --- | --- | --- |
| CIPK23-qF | CGTTTTGGAATTCGTCACTG | At1g30270 |
| CIPK23-qR | TGTTGGAAATACTTCCTCGC |  |
| SGR1 qF1 | GCGGTGGCCATTTCCTTTTA | At4g22920  a key gene in chlorophyll degradation |
| SGR1 qR1 | AGTTCCCATCTCCATGCACA |  |
| WRKY70 qF1 | CATGGATTCCGAAGATCACA | At3g56400  a negative regulator in leaf senescence |
| WRKY70 qR1 | CTGGCCACACCAATGACAA |  |
| ORE1qF1 | CTTACCATGGAAGGCTAAGATGGG | At5g39610  a positive regulator in leaf senescence |
| ORE1qR1 | TCGGGTATTTCCGGTCTCTCAC |  |
| Actin2-qF | AAGCTGGGGTTTTATGAATGG | At3g18780 |
| Actin2-qR | TTGTCACACACAAGTGCATCTA |  |
| LP | TTGTGATCCTCTTGCATAGGG | SALK_036154  Designed by http://signal.salk.edu/tdnaprimers.2.html |
| RP | AATCATCCCGGACAAAGTACC |  |
| LBb1.3 | ATTTTGCCGATTTCGGAAC | Left border primer of the T-DNA insertion  Designed by http://signal.salk.edu/tdnaprimers.2.html |
