# Supplementary figures and images for: NH4+ Toxicity, Which Is Mainly Determined by the High NH4+/K+ Ratio, Is Alleviated by CIPK23 in Arabidopsis
Source: Plants (Basel). 2020 Apr 14;9(4):501. doi: 10.3390/plants9040501 (PMC7238117; doi:10.3390/plants9040501)

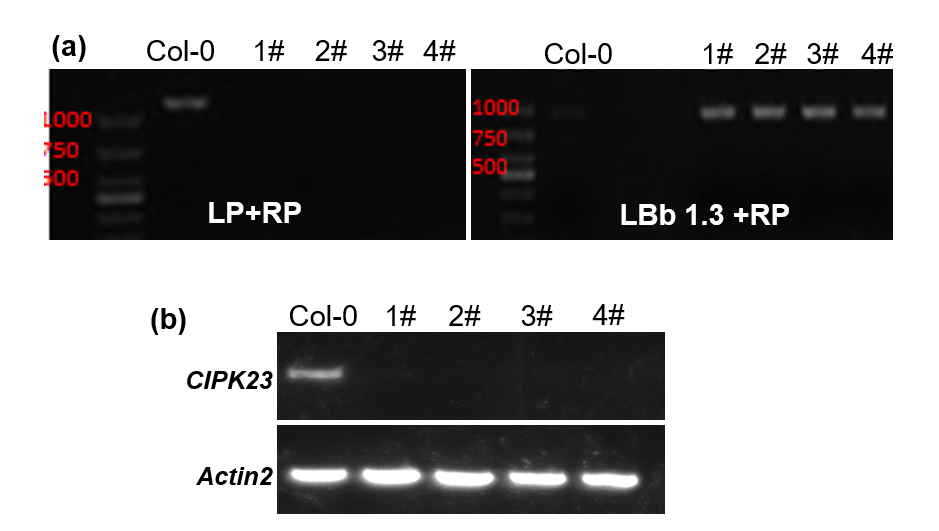

Supplement: Supplementary file 1 [file plants-09-00501-s001.zip › Figure S/Figure S1.tif]

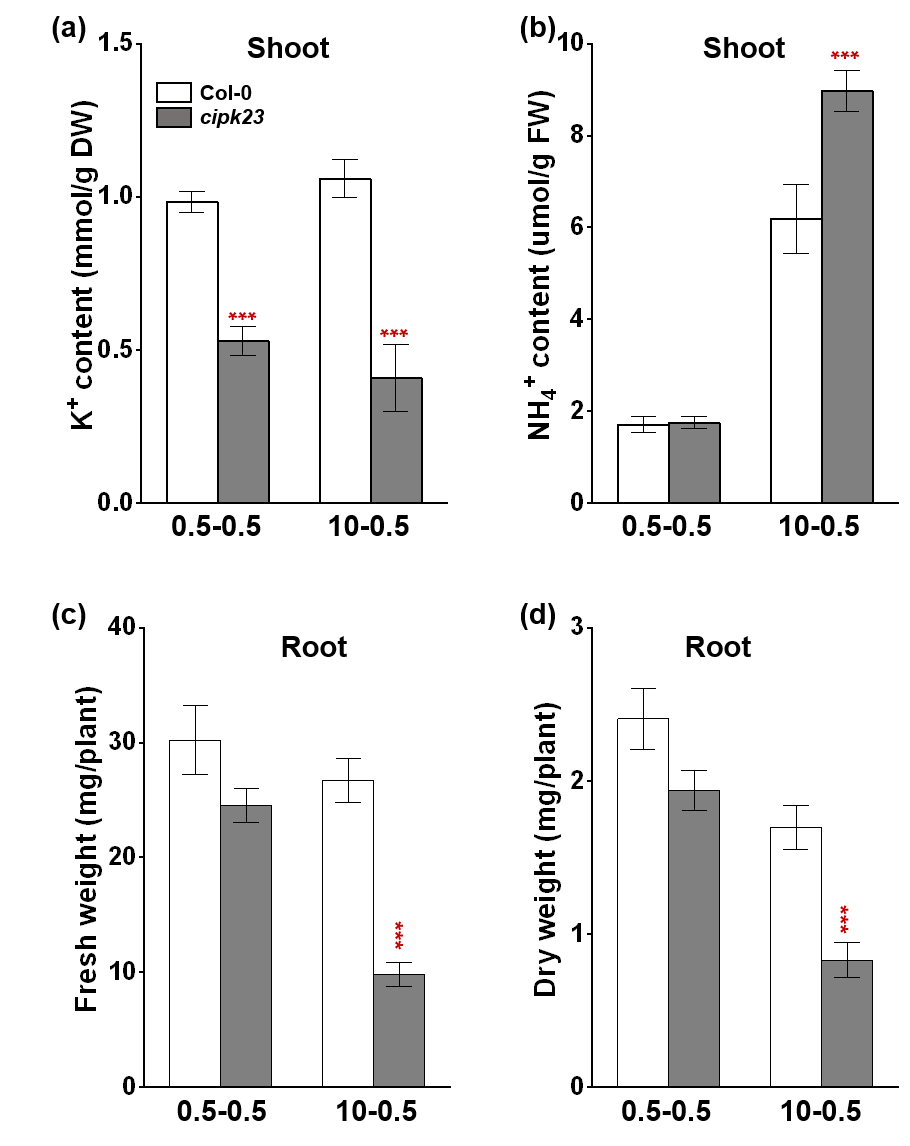

Supplement: Supplementary file 1 [file plants-09-00501-s001.zip › Figure S/Figure S2.tif]

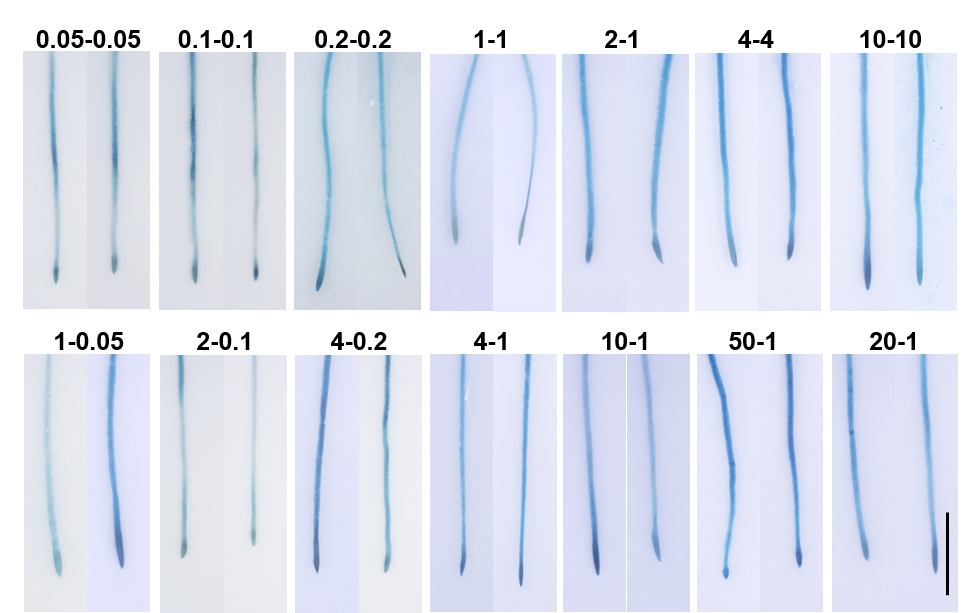

Supplement: Supplementary file 1 [file plants-09-00501-s001.zip › Figure S/Figure S3.tif]

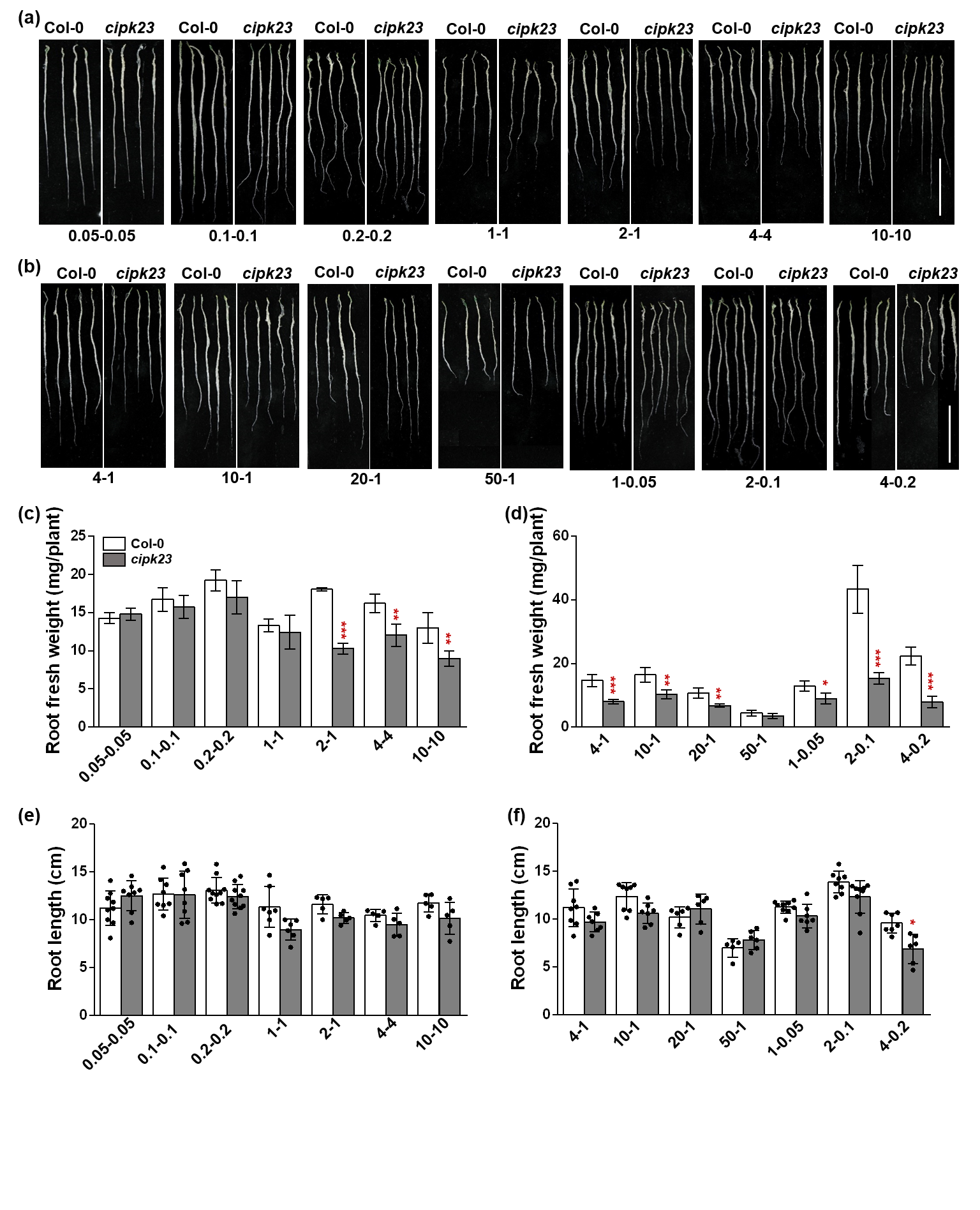

Supplement: Supplementary file 1 [file plants-09-00501-s001.zip › Figure S/Figure S4.tif]

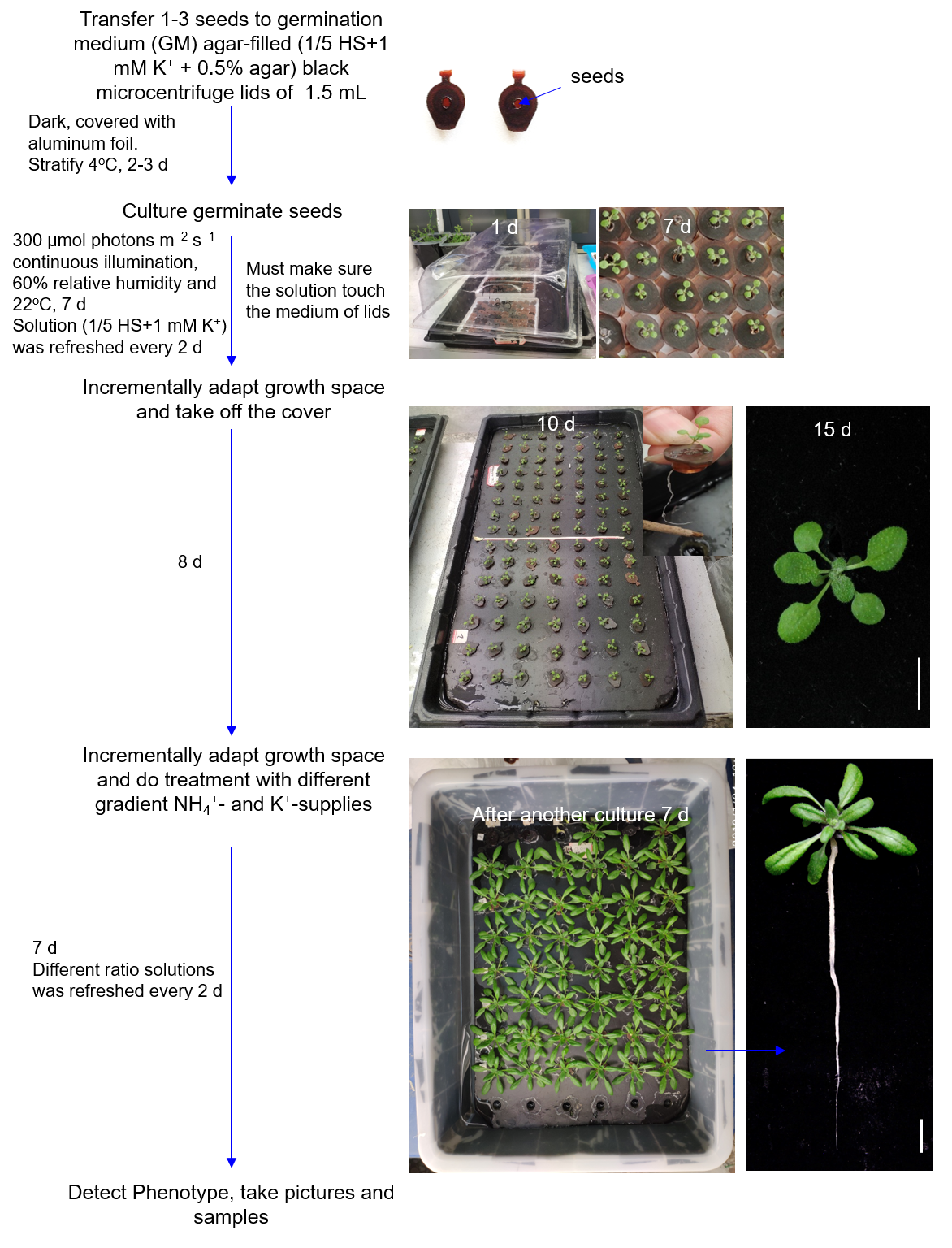

Supplement: Supplementary file 1 [file plants-09-00501-s001.zip › Figure S/Figure S5.tif]
